# The Cholesterol-Modulating Effect of Methanol Extract of Pigeon Pea (*Cajanus cajan* (L.) Millsp.) Leaves on Regulating LDLR and PCSK9 Expression in HepG2 Cells

**DOI:** 10.3390/molecules24030493

**Published:** 2019-01-30

**Authors:** Heng-Yuan Chang, Jia-Ru Wu, Wan-Yun Gao, Huei-Ru Lin, Pei-Yi Chen, Chen-I Chen, Ming-Jiuan Wu, Jui-Hung Yen

**Affiliations:** 1 School of Post-Baccalaureate Chinese Medicine, Tzu Chi University, Hualien 970, Taiwan; hychang@mail.tcu.edu.tw; 2 Department of Molecular Biology and Human Genetics, Tzu Chi University, Hualien 970, Taiwan; U8931246@yahoo.com.tw (J.-R.W.); 102712131@gms.tcu.edu.tw (W.-Y.G.); 3 Department of Laboratory Medicine and Biotechnology, Tzu Chi University, Hualien 970, Taiwan; rita0107@gms.tcu.edu.tw; 4 Center of Medical Genetics, Buddhist Tzu Chi General Hospital, Hualien 970, Taiwan; pyc571@gmail.com; 5 Crop Improvement Department, Taitung District Agricultural Research and Extension Station, Taitung 675, Taiwan; 652@mail.ttdares.gov.tw; 6 Department of Biotechnology, Chia Nan University of Pharmacy and Science, Tainan 717, Taiwan; mingjiuanwu@gmail.com

**Keywords:** *Cajanus cajan* (L.) Millsp., LDLR, PCSK9, HNF-1α, cajaninstilbene acid

## Abstract

Pigeon pea (*Cajanus cajan* (L.) Millsp.) is a legume crop consumed as an indigenous vegetable in the human diet and a traditional medicinal plant with therapeutic properties. The current study highlights the cholesterol-modulating effect and underlying mechanisms of the methanol extract of *Cajanus cajan* L. leaves (MECC) in HepG2 cells. We found that MECC increased the LDLR expression, the cell-surface LDLR levels and the LDL uptake activity in HepG2 cells. We further demonstrated that MECC suppressed the proprotein convertase subtilisin/kexin type 9 (PCSK9) mRNA and protein expression, but not affected the expression of other cholesterol or lipid metabolism-related genes including inducible degrader of LDLR (IDOL), HMG-CoA reductase (HMGCR), fatty acid synthase (FASN), acetyl-CoA carboxylase (ACC1), and liver X receptor-α (LXR-α) in HepG2 cells. Furthermore, we demonstrated that MECC down-regulated the PCSK9 gene expression through reducing the amount of nuclear hepatocyte nuclear factor-1α (HNF-1α), a major transcriptional regulator for activation of PCSK9 promoter, but not that of nuclear sterol-responsive element binding protein-2 (SREBP-2) in HepG2 cells. Finally, we identified the cajaninstilbene acid, a main bioactive stilbene component in MECC, which significantly modulated the LDLR and PCSK9 expression in HepG2 cells. Our current data suggest that the cajaninstilbene acid may contribute to the hypocholesterolemic activity of *Cajanus cajan* L. leaves. Our findings support that the extract of *Cajanus cajan* L. leaves may serve as a cholesterol-lowering agent.

## 1. Introduction

*Cajanus cajan* (L.) Millsp., commonly known as the pigeon pea, is a perennial legume crop cultivated in the sub-tropical and semi-arid tropical regions. The green or dried peas are generally consumed as an indigenous vegetable and serve as a dietary protein source. In addition to being used as a nutritional supplement, *Cajanus cajan* L. has also been used as a traditional medicinal plant [1,2]. The ethnopharmacological efficacy and biological or pharmacological activities, such as antioxidant, anti-inflammation, anti-cancer, anti-atherogenic, and hypolipidemic activities have been found in different parts of *Cajanus cajan* L. [3,4,5,6]. Chemical analyses indicated that the leaves of *Cajanus cajan* L. are rich in flavonoids and stilbenes [7,8,9]. Among them, cajaninstilbene acid (3-hydroxy-4-prenyl-5-methoxystilbene-2-carboxylic acid, CSA), a type of stilbene, is present predominantly in its leaves [10]. The stilbene-containing extract of *Cajanus cajan* L. reduced the plasma cholesterol in diet-induced hypercholesterolemic mice [11].

The level of plasma low-density lipoprotein cholesterol (LDL-C) is positively correlated with the risk of hypercholesterolemia, atherosclerosis and cardiovascular diseases [12,13,14]. The LDL receptor (LDLR) in the hepatocyte is responsible for the removal of LDL-C from the bloodstream and the maintenance of cholesterol homeostasis [15]. The plasma LDLs interact with hepatic LDLR are internalized into clathrin-coated pits through receptor-mediated endocytosis and subsequently undergo lysosomal degradation, whereas the LDLR is recycled back to the cell membrane. As a result, the abundance of LDLR plays a critical role in the maintenance of cholesterol homeostasis [16]. The enhancement of the hepatic LDLR expression or activity effectively reduced the plasma cholesterol. Moreover, the LDLR deficiency or mutation has been reported to increase plasma LDL-C levels and cause hypercholesterolemia as well as atherosclerosis [17,18].

The expression of LDLR is regulated transcriptionally and post-transcriptionally. The LDLR expression is transcriptionally activated by sterol-responsive element binding proteins (SREBPs). The functional SREBP-2 protein in the nucleus interacts with the sterol-responsive element (SRE) of the LDLR promoter and enhances the transcription of LDLR [19]. Moreover, the level of LDLR protein is downregulated post-transcriptionally by proprotein convertase subtilisin/kexin type 9 (PCSK9). The PCSK9 is an extracellular subtilisin-related serine protease that binds tightly to the LDLR, is internalized, and diverts LDLR toward lysosomal degradation, instead of recycling to the membrane [20]. PCSK9 is known to serve as a key modulator for the regulation of the plasma LDL-C. High levels of the PCSK9 protein reduce the level of LDLR protein in the hepatocytes, cause an elevation in the plasma LDL-C and increase the risk of cardiovascular disease [21].

Several studies have demonstrated that the attenuation of activity or expression of PCSK9 increases the level and LDL uptake activity of LDLR in hepatocytes. Recent studies demonstrated that monoclonal antibodies neutralized the PCSK9 protein can reduce the plasma cholesterol in patients with hypercholesterolemia [13,22,23]. In addition to neutralizing antibodies, phytochemicals such as berberine, curcumin, tanshinone IIA, and pinostrobin have been demonstrated to decrease the gene expression of PCSK9 through the regulation of transcription factors and induce hypocholesterolemic effects in hepatic cells [24,25,26,27,28]. The activity of PCSK9 promoter is regulated by transcriptional activators such as SREBP-2 and hepatocyte nuclear factor 1α (HNF-1α) [25,29]. The nuclear HNF-1α was found to bind the promoter of PCSK9 for activation of gene expression. The attenuation of the HNF-1α/PCSK9 promoter binding activity causes the reduction of the PCSK9 expression and increases the LDL uptake activity in hepatic cells [25,26].

Recently, a new pigeon pea cultivar “Taitung No. 3” has been thrived in the East Taiwan aboriginal area and is a staple foods in the villagers’ diet due to its high level of anthocyanin and antioxidant activity [5]. However, no report on the cholesterol reducing activity or underlying molecular mechanism in the leaves of this cultivar could be found. In the present study, we aim to investigate the cholesterol-modulating effect and underlying mechanisms of the methanol extract of pigeon pea leaves. Particular attention will be paid to the effects on the gene expression of LDLR and PCSK9 in HepG2 cells.

## 2. Results

### 2.1. The Effect of Methanol Extract of Cajanus cajan L. Leaves (MECC) on Cell Viability in HepG2 Cells

**Figure 1:**
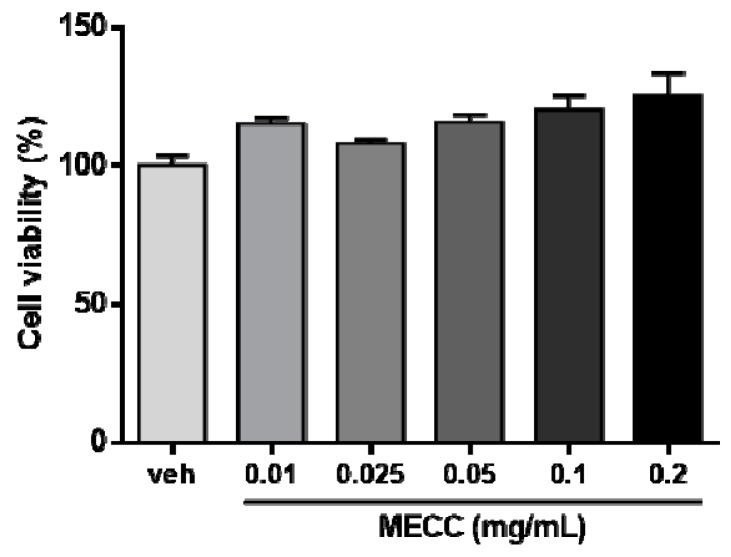
Effects of methanol extract of *Cajanus cajan* L. leaves (MECC) on cell viability in HepG2 cells. HepG2 cells were cultured in LPDS medium and treated with vehicle (0.1% DMSO) or MECC (0.01, 0.025, 0.05, 0.1 and 0.2 mg/mL) for 24 h. The cell viability was measured by MTT assay, and the data were presented as the means ± SD from three independent experiments.

To investigate the potential cholesterol-modulating effect of the methanol extract of *Cajanus cajan* L. leaves (MECC), first, we determined the cytotoxic effects of MECC on HepG2 cells. Cells were treated with vehicle (0.1% DMSO) or MECC (0.01–0.2 mg/mL) for 24 h in DMEM medium with 10% lipoprotein-deficient serum (LPDS) and the cell viability was measured using MTT assay. As shown in Figure 1, treatment of MECC (0.01–0.2 mg/mL) had no cytotoxic effect on HepG2 cells.

### 2.2. The Effect of MECC on the LDLR Expression in HepG2 Cells

**Figure 2:**
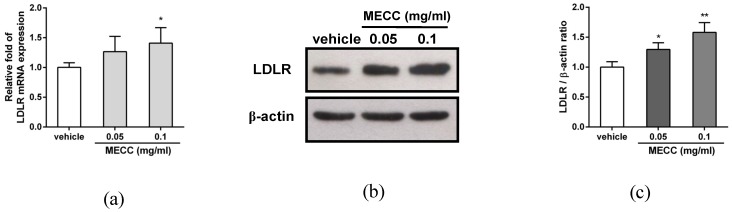
Effects of MECC on the LDLR expression in HepG2 cells. HepG2 cells were cultured in LPDS medium and treated with vehicle (0.1% DMSO) or MECC (0.05 and 0.1 mg/mL) for 24 h. (**a**) The level of LDLR mRNA was measured by reverse transcription real-time PCR analysis. (**b**) The levels of LDLR and β-actin proteins were analyzed by Western blot analysis. The experiments were replicated three times, and a representative blot was shown. (**c**) The intensity of LDLR normalized to the β-actin protein is presented as the means ± SD of three independent experiments. * *p* < 0.05 and ** *p* < 0.01 represent significant differences compared to the vehicle-treated group.

The elevation of the hepatic LDLR protein is known to decrease the level of plasma LDL-C. Therefore, the effect of MECC on the LDLR gene expression was investigated in HepG2 cells. Cells were treated with vehicle or MECC (0.05 and 0.1 mg/mL) for 24 h, and then the LDLR mRNA and protein expression was determined using reverse transcription real-time PCR and Western blot analysis. As shown in Figure 2a, MECC (0.05 and 0.1 mg/mL) increased the level of LDLR mRNA expression by approximately 1.2- and 1.4-fold compared with the vehicle control. The cellular LDLR protein was significantly increased in the MECC-treated HepG2 cells by approximately 1.3- and 1.6-fold as compared with the vehicle group (*p* < 0.05 and *p* < 0.01), respectively (Figure 2b,c). These results suggest that MECC increased the LDLR expression in HepG2 cells.

**Figure 3:**
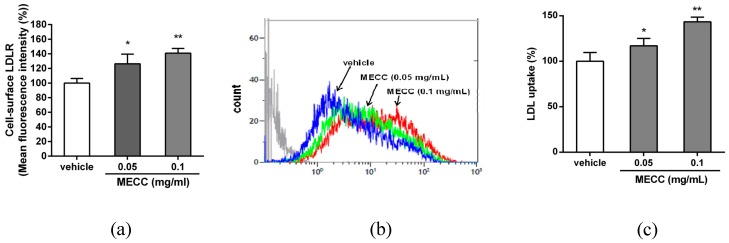
Effects of MECC on the level of cell-surface LDLR and LDL uptake activity in HepG2 cells. HepG2 cells were cultured in LPDS medium and treated with vehicle (0.1% DMSO) or MECC (0.05 and 0.1 mg/mL) for 24 h. (**a**) The amount of cell-surface LDLR was measured by flow cytometry analysis. (**b**) The cells were incubated with BODIPY^®^-FL-LDL for 24 h, and the LDL uptake was determined using flow cytometry analysis. The experiments were replicated three times, and a representative histogram was shown. (**c**) Summary of LDL uptake. The data are presented as the means ± SD from three independent experiments. * *p* < 0.05 and ** *p* < 0.01 represent significant differences compared to the vehicle-treated group.

Furthermore, we examined the amount of cell-surface LDLR in the MECC-treated HepG2 cells by flow cytometry analysis. As shown in Figure 3a, the MECC (0.05 and 0.1 mg/mL) significantly increased the levels of cell-surface LDLR on HepG2 cells by approximately 22% and 40%, compared to the vehicle-treated cells (*p* < 0.05 and *p* < 0.01), respectively. To investigate whether cell-surface LDLR increase is associated with enhancement of its activity, we further evaluated the effect of MECC on the LDL uptake in HepG2 cells. As shown in Figure 3b,c, cells treated with MECC (0.05 and 0.1 mg/mL) significantly increased the amount of LDL uptake by approximately 17% and 43% compared to the vehicle group (*p* < 0.05 and *p* < 0.01), respectively. This data demonstrated that the MECC increases the LDLR protein as well as LDL uptake in hepatic cells. These above results reveal that MECC enhanced the hepatic LDLR expression and activity.

### 2.3. The Effect of MECC on the mRNA Expression of Selected Genes for Cholesterol Homeostasis and Lipogenesis

**Figure 4:**
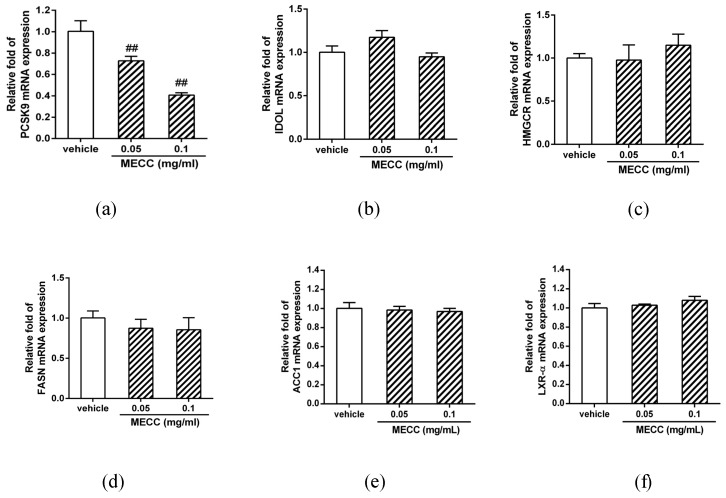
Effects of MECC on the expression of cholesterol homeostasis- and lipogenesis-related genes. HepG2 cells were cultured in LPDS medium and treated with vehicle (0.1% DMSO) or MECC (0.05 and 0.1 mg/mL) for 24 h. Cellular RNA was prepared, and the mRNA expression of the (**a**) PCSK9 (**b**) IDOL (**c**) HMGCR (**d**) FASN (**e**) ACC1 and (**f**) LXR-α was determined by reverse transcription real-time PCR. The data are presented as the means ± SD from three independent experiments. ## *p* < 0.01 represent significant differences compared to the vehicle-treated group.

We further investigated whether MECC altered the mRNA expression of selected genes for cholesterol homeostasis and lipogenesis by reverse transcription real-time PCR analysis. First, we analyzed the genes involved in the LDLR modulation and cholesterol homeostasis in hepatic cells including PCSK9 [30], inducible degrader of LDLR (IDOL) [31] and HMG-CoA reductase (HMGCR). As shown in Figure 4a, MECC (0.05 and 0.1 mg/mL) markedly decreased the level of PCSK9 mRNA by approximately to 0.78- and 0.46-fold as compared with the vehicle-treated group (*p* < 0.01), respectively. However, the MECC-treated cells did not exhibit changes in the mRNA expression of IDOL and HMGCR (Figure 4b,c). Next, the effect of MECC on the genes involved in lipogenesis was also evaluated. The mRNA expression of fatty acid synthase (FASN) was slightly but not significantly decreased in the MECC-treated cells (Figure 4d). The expression levels of acetyl-CoA carboxylase (ACC1) and liver X receptor-α (LXRα) were not altered in the MECC-treated cells (Figure 4e,f).

### 2.4. The Effect of MECC on the PCSK9 Protein Expression in HepG2 Cells

**Figure 5:**
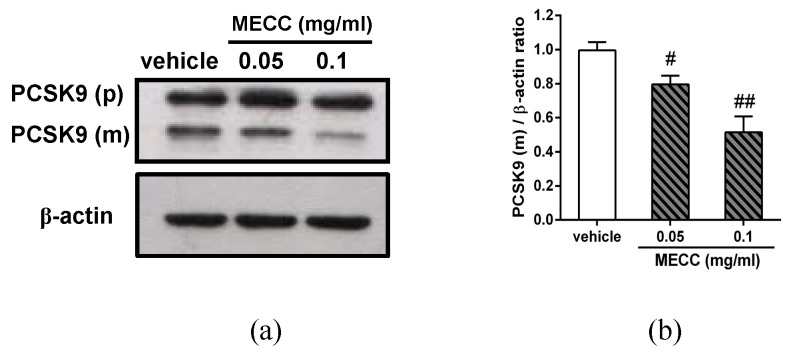
Effects of MECC on PCSK9 protein expression in HepG2 cells. Cells were cultured in LPDS medium and treated with vehicle (0.1% DMSO) or MECC (0.05 and 0.1 mg/mL) for 24 h. (**a**) The PCSK9 proprotein form (p), mature form (m) and β-actin proteins were determined by Western blot analysis. The experiments were at least replicated three times, and a representative blot was shown. (**b**) The intensity of the PCSK9 mature form protein (PCSK9 (m)) normalized to the β-actin is presented as the means ± SD of three independent experiments. # *p* < 0.05 and ## *p* < 0.01 represent significant differences compared to the vehicle-treated group.

It is known that the cell-surface LDLR was negatively regulated by the mature form of PCSK9 protein [32]. The above results showed that MECC significantly decreased PCSK9 mRNA expression. We further investigated the effect of MECC on the PCSK9 protein in HepG2 cells. As shown in Figure 5a,b, HepG2 cells treated with MECC (0.05 and 0.1 mg/mL) significantly reduced the level of mature PCSK9 protein (PCSK9 (m)) by approximately to 0.80- and 0.51-fold as compared with the vehicle-treated cells (*p* < 0.05 and *p* < 0.01), respectively, but did not significantly change that of proprotein (PCSK9 (p)). These results indicated that the MECC reduced the mature form of PCSK9, which resulted in the increases of the levels of LDLR protein and activity in HepG2 cells.

### 2.5. The Effect of MECC on Regulation of the PCSK9 Promoter and Transcriptional Activators in HepG2 Cells

**Figure 6:**
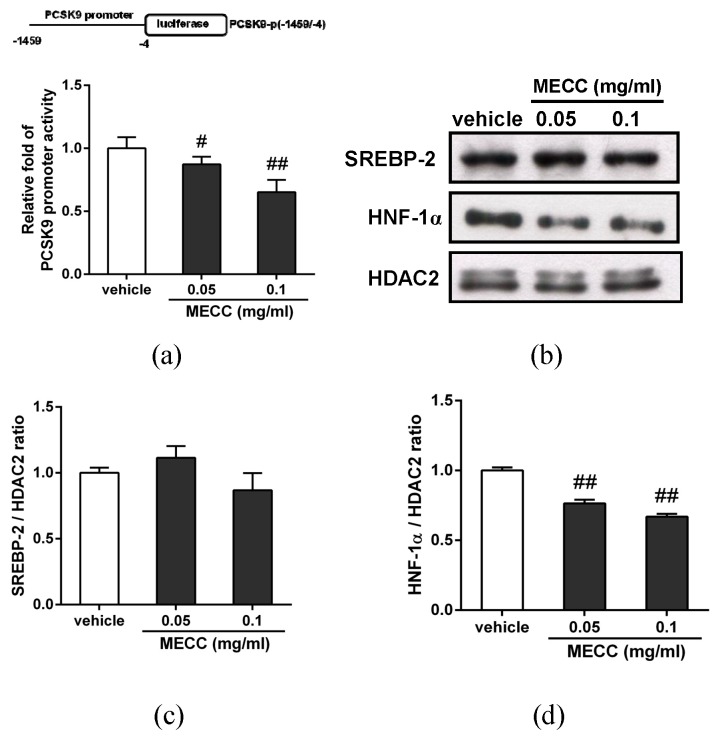
Effects of MECC on the PCSK9 promoter activity and transcriptional activators in HepG2 cells. (**a**) HepG2 cells were transfected with PCSK9 promoter-luciferase reporter plasmid (PCSK9-p(-1459/-4)) and *Renilla* control plasmid for 24 h, and treated with vehicle or MECC (0.05 and 0.1 mg/mL) for an additional 24 h. The luciferase activity was measured and normalized to the respective *Renilla* luciferase activity. The data are presented as the means ± SD from three independent experiments. (**b**) The nuclear SREBP-2, HNF-1α and HDAC2 proteins were determined by Western blot analysis. The experiments were at least replicated three times, and a representative blot was shown. The normalized intensities of SREBP-2 (**c**) and HNF-1α (**d**) versus HDAC2 are presented as the means ± SD of three independent experiments. # *p* < 0.05 and ## *p* < 0.01 represent significant differences compared to the vehicle-treated group.

To elucidate the possible mechanism by which MECC reduced the PCSK9 gene expression, we further analyzed the effect of MECC on the PCSK9 promoter activity in HepG2 cells. The cells were transfected with the PCSK9 promoter-luciferase reporter plasmid (PCSK9-p(-1459/-4)) for 24 h, followed by treatment with the vehicle or MECC (0.05 and 0.1 mg/mL) for an additional 24 h. The luciferase activity data showed that the PCSK9 promoter activity was markedly decreased in the MECC (0.05 and 0.1 mg/mL)-treated cells (Figure 6a). This data showed that MECC reduces the PCSK9 gene expression via the inhibition of the promoter activity in hepatic cells.

Transcriptional factors such as sterol regulatory element-binding protein 2 (SREBP-2) and hepatocyte nuclear factor-1α (HNF-1α) are critical for the transcriptional activation of PCSK9 gene expression [25]. To examine whether these transcriptional activators are involved in the MECC-mediated PCSK9 reduction, we further investigated the effect of MECC on the nuclear SREBP-2 and HNF-1α proteins in HepG2 cells. The cells were treated with MECC (0.05 and 0.1 mg/mL), and the levels of these transcription factors in nucleus were determined by Western blot analysis. As shown in Figure 6b,c, MECC did not alter the level of nuclear SREBP-2 in HepG2 cells. The amount of nuclear HNF-1α in MECC-treated cells was significantly reduced by approximately 23% and 38% compared to the vehicle-treated cells, respectively (*p* < 0.01) (Figure 6b,d). These data suggested that MECC decreased the level of nuclear HNF-1α protein, which may cause to down-regulate the PCSK9 gene expression in HepG2 cells.

### 2.6. Measurement of the Main Bioactive Components in MECC

It has been reported that the four bioactive antioxidant components including cajaninstilbene acid, pinostrobin, vitexin and orientin (Figure S1) are rich in the *Cajanus cajan* L. leaves [4]. Therefore, we further determined the contents of the four main components in the MECC using liquid chromatography tandem-mass spectrometry (LC-MS/MS) analysis as described in Materials and Methods. As shown in Figure S1 and Table 1, we found that the contents of cajaninstilbene acid, pinostrobin, vitexin and orientin in the MECC are approximately 0.23 mg/g extract, 0.01 mg/g extract, 0.04 mg/g extract, and 0.13 mg/g extract, respectively.

**Figure 7:**
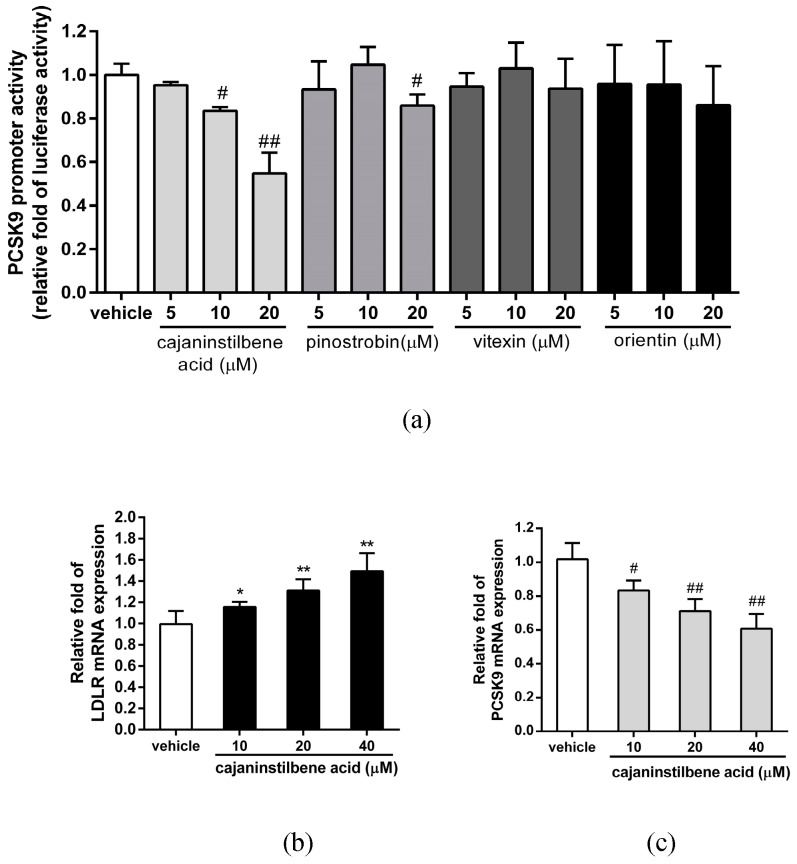
Effects of cajaninstilbene acid on LDLR and PCSK9 mRNA expression in HepG2 cells. (**a**) The PCSK9 promoter/luciferase reporter stable cells were treated with vehicle (0.1% DMSO), cajaninstilbene acid, pinostrobin, vitexin or orientin (5–20 μM) for 24 h. The luciferase activity was measured and normalized to respective protein concentration. The data are presented as the means ± SD from three independent experiments. (**b**) HepG2 cells were cultured in LPDS medium and treated with vehicle (0.1% DMSO) or cajaninstilbene acid (10–40 μM) for 24 h. LDLR mRNA expression was measured using reverse transcription real-time PCR analysis. The data are presented as the means ± SD from three independent experiments. * *p* < 0.05 and ** *p* < 0.01 represent significant differences compared to the vehicle-treated group. (**c**) PCSK9 mRNA expression was measured using reverse transcription real-time PCR analysis. The data are presented as the means ± SD from three independent experiments. # *p* < 0.05 and ## *p* < 0.01 represent significant differences compared to the vehicle-treated group.

Furthermore, we examined the effect of cajaninstilbene acid, pinostrobin, vitexin, and orientin on the transcriptional activity of PCSK9 promoter in the PCSK9 promoter/luciferase reporter stable cell line. The activity of PCSK9 promoter was significantly inhibited by the cajaninstilbene acid (10 and 20 μM) and slightly reduced by pinostrobin (20 μM), however, was not altered by vitexin and orientin in hepatic cells (Figure 7a). We further determined whether cajaninstilbene acid is involved in the modulation of LDLR and PCSK9 gene expression. As shown in Figure 7b,c, cajaninstilbene acid (10–40 μM) significantly increased the LDLR mRNA expression, and reduced the PCSK9 expression in HepG2 cells. These results indicate that the cajaninstilbene acid may play a critical role on the MECC-mediated modulation of LDLR and PCSK9 expression.

## 3. Discussion

Phenolic phytochemicals are the most abundant anti-oxidants in the diet, and there may be a causal relationship between polyphenol consumption and improvements in cardiovascular function [33,34,35]. The extract of pigeon pea (*Cajanus cajan* L.) leaves has been demonstrated to contain a number of polyphenols such as flavonoids and stilbenes, which possess the strongest anti-oxidative and anti-inflammatory activities [4,7]. The level of phytochemicals varies among different cultivars and harvest times, and results in different bioactivities [10]. In this study, we demonstrated for the first time that the methanol extract of pigeon pea cultivar “Taitung No. 3” leaves (MECC) exerts dual-modulating effects, increasing the gene expression of LDLR and reducing that of PCSK9, resulting in the elevation of cell-surface LDLR and its LDL uptake activity in HepG2 cells. We also demonstrated that MECC down-regulated PCSK9 expression through the attenuation of nuclear HNF-1α, but not SREBP-2, in hepatic cells. Furthermore, we have identified cajaninstilbene acid as a main component in MECC, which may contribute to modulate the LDLR and PCSK9 expression in HepG2 cells.

Hepatic LDLR removes more than 70% of the LDL-C from circulation and modulates the plasma cholesterol homeostasis. The increases in the amounts of LDLR or cell-surface LDLR activity resulted in promoting the clearance of LDLs [16]. The levels of hepatic LDLR protein are regulated at both the transcriptional and post-transcriptional levels. In the present study, we demonstrated that MECC increased the LDLR mRNA expression and elevated the levels of total and cell-surface LDLR proteins, which in turn increased the LDLR activity for removing LDLs by hepatocytes. The LDLR transcription is known to stimulate by nuclear SREBP-2 in the hepatic cells. In our study, MECC increased the amount of LDLR mRNA expression, however, MECC did not change the nuclear SREBP-2 level in HepG2 cells. These data suggest MECC may activate the SREBP-2-independent regulation for LDLR transcription in hepatic cells. In addition to transcriptional regulation, the mRNA stability also plays a critical role for post-transcriptional maintenance of protein amount. Whether other transcriptional regulators such as SP1 which has been shown to modulate the LDLR transcription [36] is induced or the mRNA stability is prolonged in MECC-treated hepatic cells remains unclear and needs to be further clarified.

Recent studies demonstrated that the LDLR was post-translationally modified by PCSK9 and inducible degrader of LDLR (IDOL) proteins for targeting to lysosomal degradation. PCSK9 gene knockout mice and loss-of-function PCSK9 mutations in humans were reported to have the cholesterol-lowering effects in the bloodstream [37,38,39]. Mature form of PCSK9 proteins in circulation bind to the hepatic cell-surface LDLR, and these LDLR/PCSK9 protein complexes were delivered to the endosome/lysosome for degradation. Therefore, inhibition of PCSK9 function by monoclonal antibodies or gene expression by small molecules is a promising strategy for hypercholesterolemia therapy [40,41]. In addition to PCSK9, the IDOL, an E3-ubiqutin ligase can promote LDLR protein ubiquitination and degradation. Thus, the IDOL protein can also serve as a novel post-transcriptional modulator for the level of LDLR in hepatic cells [42,43]. In this study, MECC significantly reduced the expression of PCSK9 mRNA, but the IDOL expression was not changed in HepG2 cells. Furthermore, we demonstrated MECC inhibited the PCSK9 promoter activity and identified MECC reduced the amount of nuclear HNF-1α protein, but does not alter the nuclear SREBP-2 level. These findings suggest that MECC-mediated PCSK9 down-regulation was regulated by reduction of nuclear HNF-1α, however, independence of the activation of SREBP-2. Moreover, we found that MECC did not change the expression of HMG-CoA reductase (HMGCR), which is a critical enzyme for cholesterol biosynthesis in hepatic cells. This suggested the modulation of the LDLR and PCSK9 gene expression by MECC is not due to the suppression of cholesterol biosynthesis in hepatic cells. Our current results also showed that the expression of lipogenesis-related genes, including FASN, ACC1 and LXR-α, were not significantly changed in MECC-treated HepG2 cells. Our findings support MECC down-regulated PCSK9 gene expression, which resulted in increases of LDLR proteins in hepatic cells. The leaves of *Cajanus cajan* L. possess potential cholesterol-lowering activity for regulation of lipid homeostasis.

The precursor form of hepatic PCSK9 is processed in the endoplasmic reticulum by auto-cleavage at the C-terminal of the proprotein to form a mature protease that is secreted into the bloodstream [44,45]. In the present study, MECC markedly reduced mature form of PCSK9 protein. However, a reduction of PCSK9 proprotein did not occurr in MECC-treated HepG2 cells. Our current data suggest that MECC has the potential to inhibit the auto-catalytic activity of PCSK9 for regulation of the proprotein processing pathway. The detailed mechanisms involved in the modulation of auto-catalytic activity of PCSK9 protein by MECC need to be further investigated.

The extract of *Cajanus cajan* L. leaves has been demonstrated to contain a number of polyphenols, such as cajaninstilbene acid, pinostrobin, vitexin, and orientin, which possess the strongest anti-oxidative and anti-inflammatory activities [4]. In the present study, we demonstrated that cajaninstilbene acid is the most abundant component of four main anti-oxidants in MECC. Cajaninstilbene acid is an active stilbene constituent present in *Cajanus cajan* L. leaves and possesses several pharmacological effects including anti-oxidant, anti-inflammatory, neuroprotective, and anti-cancer activities in cell or animal models [46,47,48]. Our current data demonstrated cajaninstilbene acid can up-regulate LDLR and down-regulate PCSK9 expression in hepatic cells. These findings suggest that cajaninstilbene acid is the principal bioactive compound in MECC for modulation of LDLR and PCSK9 gene expression. In addition to cajaninstilbene acid, whether other stilbenes or flavonoids presented in MECC with similar activities or the synergistic effect of these compounds may contribute to modulate LDLR and PCSK9 expression for cholesterol homeostasis needs to be further investigated.

## 4. Materials and Methods

### 4.1. Plant Materials and Chemicals

**Figure 8:**
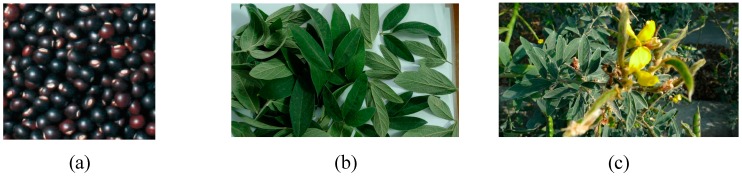
Representative *Cajanus cajan* (L.) Millsp. plants cultivar Taitung No. 3; (**a**) mature pea; (**b**) sample leaves; and (**c**) the early pod bearing stage harvest on 121 days after sowing.

Pigeon pea *(Cajanus cajan* (L.) Millsp.) cultivar Taitung No. 3 has a dark purple seed coat due to high levels of cyanidin-3-glucoside and peonidin-3-glucoside [5] (Figure 8a). The plants were sown in July in Taitung District Agricultural Research and Extension Station (Taitung, Taiwan) and leaves were harvested in mid-January next year (Figure 8b) when plants were in the early pod bearing stage (Figure 8c). This is the stage that active components and antioxidant activity reached high levels [10]. The voucher specimen (CC001) was deposited in Crop Improvement Department, Taitung District Agricultural Research and Extension Station. Cajaninstilbene acid was kindly provided by Dr. Chin-Piao Chen (Tzu Chi University of Science and Technology, Hualien, Taiwan). G418 sulfate was purchased from Amresco, Inc. (Solon, OH, USA). Pinostrobin, vitexin, orientin, RPMI-1640 medium, non-essential amino acids (NEAA), dimethyl sulfoxide (DMSO), and other chemicals unless otherwise indicated were purchased from Sigma-Aldrich Co. (St. Louis, MO, USA).

### 4.2. Methanol Extraction of the Leaves of Pigeon Pea (Cajanus cajan (L.) Millsp.)

The leaves of pigeon pea *(Cajanus cajan* (L.) Millsp.) cultivar Taitung No. 3 were cut into small pieces, dried and powdered. The dried powder (3 kg) was extracted with methanol (20 L × 3), and the extract was combined and concentrated *in vacuo* to yield a brown syrup (230 g). The syrup (200 mg) was dissolved in 1 mL of dimethyl sulfoxide (DMSO) and denoted as the methanol extract of *Cajanus cajan* L. leaves (MECC).

### 4.3. Cell Culture and Treatment of Methanol Extract of Cajanus cajan Leaves

HepG2 cells were obtained from the Bioresource Collection and Research Center (Hsinchu, Taiwan) and cultured in DMEM medium supplemented with 10% fetal bovine serum (FBS) (Thermo Fisher Scientific, Inc., Rockford, IL, USA) and 1% non-essential amino acids (NEAA). The cells were seeded in culture vessels for 24 h and then changed to DMEM medium supplemented with 10% lipoprotein-deficient serum (LPDS) overnight [26]. The cells were then treated with vehicle (0.1% DMSO), MECC or compounds for an additional 24 h and incubated in an incubator at 37 °C in 5% CO_2_.

### 4.4. Determination of Cell Viability by MTT Assay

The MTT assay was used for the measurement of the cell viability as previously described [49]. The cells were treated with 1 mg/mL MTT reagent and incubated at 37 °C for 4 h. The mitochondria-dependent reduction of MTT produced purple formazan crystals that were dissolved in DMSO, and the absorbance was read at 550 nm.

### 4.5. RNA Extraction and Reverse Transcription Real-Time PCR Analysis

The total cellular RNA was prepared using the Total RNA mini Kit (Geneaid, Taipei, Taiwan). Reverse transcription was carried out using the High Capacity cDNA reverse transcription kit (Thermo Fisher Scientific). Real-time PCR was performed in a reaction containing cDNA, gene-specific primers (Table 2) and the Maxima SYBR Green/ROX qPCR Master Mix (Thermo Fisher Scientific). The PCR amplification was performed in a Roche LightCycler^®^-480 Real-Time PCR System (Roche Diagnostics, Indianapolis, IN, USA) with the following conditions: 58 °C for 2 min, 94 °C for 4 min, and 40 cycles of 94 °C for 1 min, 58 °C for 1 min and 72 °C for 1 min. The target mRNA expression was measured using the ∆∆Ct method and normalized to the expression level of glyceraldehyde-3-phosphate dehydrogenase (GAPDH) in the same samples.

### 4.6. Western Blot Analysis

For preparation of the total cellular proteins, the vehicle or MECC-treated cells were harvested using RIPA buffer (Thermo Fisher Scientific). For preparation of the nuclear proteins, the cells were harvested using NE-PER nuclear and cytoplasmic extraction reagent (Thermo Fisher Scientific) according to the manufacturer’s instructions. The proteins were separated by 10% SDS-PAGE and transferred to a PVDF membrane (PerkinElmer, Boston, MA, USA). The membranes were incubated with the primary antibodies: anti-PCSK9, anti-HDAC2 (GeneTex, Irvine, CA, USA), anti-LDLR (Novus Biologicals, Littleton, CO, USA), anti-N-terminus of SREBP-2 (Cayman, Ann Arbor, MI, USA), anti-HNF-1α (Cell Signaling Technology, Danvers, MA, USA) and anti-β-actin (Sigma-Aldrich). The membranes were further incubated with the horseradish peroxidase-conjugated secondary antibodies (Santa Cruz Biotechnology) and were detected by the ECL™ Prime Western Blotting Detection Reagent and the signal was visualized on Hyperfilm™ ECL (GE Healthcare, Buckinghamshire, UK).

### 4.7. Detection of the Level of Cell-Surface LDLR

The level of cell-surface LDLR was measured by flow cytometry analysis as previously described [27]. Briefly, after the treatment of vehicle or MECC for 24 h, the cells were detached and incubated with blocking reagent (1XPBS with 5% bovine serum albumin) for 30 min. The cells were incubated with anti-LDLR antibody at 37 °C for 1 h and were then incubated with Alexa Fluor 488-conjugated goat anti-rabbit IgG (Thermo Fisher Scientific, Rockford, IL, USA) at room temperature for 30 min and detected by flow cytometry (FACScan, BD Biosciences, San Jose, CA, USA). The data were calculated using Cell Quest Pro software (version 6.0, BD Biosciences), and the level of cell-surface LDLR was expressed as the relative percentage of the geometric mean fluorescence intensity.

### 4.8. Detection of LDL Uptake

The LDL uptake was determined as previously described [27]. Briefly, the HepG2 cells were treated with the vehicle or MECC for 24 h. The medium was removed, replaced with serum-free DMEM medium and treated with BODIPY^®^ FL low-density lipoprotein (LDL) (10 μg/mL) (Thermo Fisher Scientific) at 37 °C for 24 h. The cells were analyzed by flow cytometry and data were acquired from 10,000 cells (counts). The activity of LDL uptake by cell-surface LDLR was expressed as the relative percentage of the geometric mean fluorescence intensity.

### 4.9. Transfection, Luciferase Reporter Assay and Estabishment of PCSK9 Promoter/Luciferase Reporter Stable Cell Line

The cells were co-transfected with PCSK9 promoter/luciferase reporter plasmids (PCSK9-p(-1459/-4)) [26] and *Renilla* luciferase internal control plasmids (Promega, Madison, WI, USA) using the Lipofectamine Transfection Reagent (ThermoFisher Scientific) according to the manufacturer’s instructions. After 24 h of transfection, the cells were treated with the vehicle or extracts for an additional 24 h. The luciferase activities were measured using the Dual-Luciferase Reporter Assay System Kit (Promega) according to the manufacturer’s instructions. The luciferase activities were normalized to the *Renilla* luciferase activity.

For establishment of PCSK9 promoter/luciferase reporter stable cell line, the pRMT-Luc reporter vector (OriGene Technologies, Inc., Rockville, MD, USA) containing DNA fragment of PCSK9 promoter (nucleotides -1459 to -4) [26] was constructed and transfected into Huh 7 cells using the Lipofectamine Transfection Reagent. These transfected cells were treated with G418 sulfate (600 μg/mL) and the antibiotic-resistant cell clones were selected for continuously expressing luciferase gene driven by PCSK9 promoter. The luciferase activities were measured using the Luciferase Assay System (Promega) according to the manufacturer’s instructions. The cellular protein concentration was measured using Bio-Rad Protein Assay Dye Reagent (Bio-Rad Laboratories, Hercules, CA, USA) according to the manufacturer’s instructions. The luciferase activities were normalized to the protein concentration.

### 4.10. Identification of Compounds by Liquid Chromatography-Tandem Mass Spectrometry (LC-MS/MS) Analysis

A Finnigan TSQ Quantum triple-quadrupole mass spectrometer (Thermo Fisher Scientific, USA) coupled with the Accela UPLC system (Thermo Fisher Scientific) was used for the identification of compounds in the MECC. The samples were injected into a superficially porous particle column (Agilent Poroshell 120 EC-C_18_, 2.1 mm × 100 mm, 2.7 μm) (Agilent Technology Inc, Santa Clara, CA, USA) for the liquid chromatographic (LC) separation. The column temperature was set at 40 °C. The chromatographic separation was achieved through gradient elution at a flow rate of 0.32 mL/min. Water and methanol containing 0.1% formic acid were mobile phases A and B, respectively. The following optimized chromatographic gradient was used: 20% of component B at injection was increased linearly to 25% B over the first 3 min, then to 50% B at 4 min, and then to 100% B at 4.2 min; this gradient was maintained for 1 min. The gradient returned to the initial condition at 5.7 min and was equilibrated for 2 min; the total run time was 7.7 min. Positive ionization mode was applied, and nitrogen was used as the sheath and auxiliary gases at optimal values of 30 and 5 units (arbitrary units), respectively. The analysis was performed using a spray voltage of 4000 V, and the heated capillary temperature was 270 °C. The argon collision gas pressure was set to 1.0 mTorr. To optimize the multiple reaction monitoring (MRM) conditions for the individual analytes, the post-column infusion of the standard solution was performed using Quantum TuneMaster software (Thermo Fisher Scientific). The scan time for each transition was set to 15 ms.

### 4.11. Statistical Analysis

All experiments were repeated at least three times, and the values were expressed as the mean ± SD. The results were analyzed using one-way ANOVA with Dunnett’s post hoc test, and a *p* value < 0.05 was considered significant.

## 5. Conclusions

The results in the current study demonstrated the hypocholesterolemic effect of the methanol extract of *Cajanus cajan* L. cultivar “Taitung No. 3” leaves in hepatic cells. Our current findings reveal that the methanol extract of *Cajanus cajan* L. leaves reduced nuclear HNF-1α protein and resulting in suppression of PCSK9 gene expression, which causes to increase LDLR level and LDL uptake activity in hepatic cells. The up-regulation of the LDLR as well as down-regulation of PCSK9 expression by cajaninstilbene acid may contribute to the cholesterol-modulating effect of the *Cajanus cajan* L. leaves. Our current findings provide a molecular mechanism of *Cajanus cajan* L. leaves for cholesterol homeostasis and support that the *Cajanus cajan* L. leaves extract may serve as a cholesterol-lowering agent.

## Figures and Tables

**Table 1:** Measurement of the main bioactive components in MECC.

	Cajaninstilbene Acid (mg/g Extract)	Pinostrobin (mg/g Extract)	Vitexin (mg/g Extract)	Orientin (mg/g Extract)
MECC	0.23 ± 0.04	0.01 ± 0.001	0.04 ± 0.005	0.13 ± 0.01

**Table 2:** Gene-specific primer pairs used in real-time PCR.

Genes	Primers
LDLR	5′-AGTTGGCTGCGTTAATGTGA-3′
	5′-TGATGGGTTCATCTGACCAGT-3′
PCSK9	5′-GCTGAGCTGCTCCAGTTTCT-3′
	5′-AATGGCGTAGACACCCTCAC-3′
IDOL	5′-AAGTTCTTCGTGGAGCCTCA-3′
	5′-ACTGAGTTCCACTGCCTGCT-3′
HMGCR	5′-TGATTGACCTTTCCAGAGCAAG-3′
	5′-CTAAAATTGCCATTCCACGAGC-3′
ACC1	5′-GAGGGAAGGGAATTAGAA-3′
	5′-ATCACCCCAGGGAGATAC-3′
FASN	5′-TATGCTTCTTCGTGCAGCAGTT-3′
	5′-GCTGCCACACGCTCCTCTAG-3′
LXR-α	5′-GCCGAGTTTGCCTTGCTCA-3′
	5′-TCCGGAGGCTCACCAGTTTC-3′
GAPDH	5′-ATGAGAAGTATGACAACAGCCT-3′
	5′-AGTCCTTCCACGATACCAAAGT-3′

## Supplementary Materials

The following are available online, Figure S1. Chemical structure of (a) cajaninstilbene acid; (b) pinostrobin; (c) vitexin; and (d) orientin. (e) The multiple reaction monitoring (MRM) chromatograms of standard solution with 2.5 μM of cajaninstilbene acid, pinostrobin, vitexin, and orientin. (**f**) The MRM chromatograms of 10 μL MECC sample (50 mg/mL).
