# Supplementary material for: The Cholesterol-Modulating Effect of Methanol Extract of Pigeon Pea (Cajanus cajan (L.) Millsp.) Leaves on Regulating LDLR and PCSK9 Expression in HepG2 Cells
Source: Molecules. 2019 Jan 30;24(3):493. doi: 10.3390/molecules24030493 (PMC6385019; doi:10.3390/molecules24030493)
Supplement: Supplementary file 1 [file molecules-24-00493-s001.pdf]

Supplemental Figures

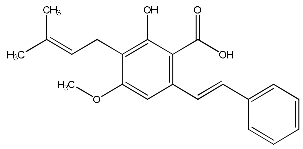

(**a**)

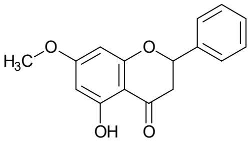

(**b**)

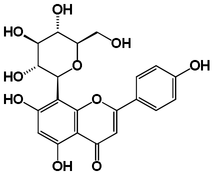

(**c**)

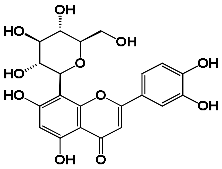

(**d**)

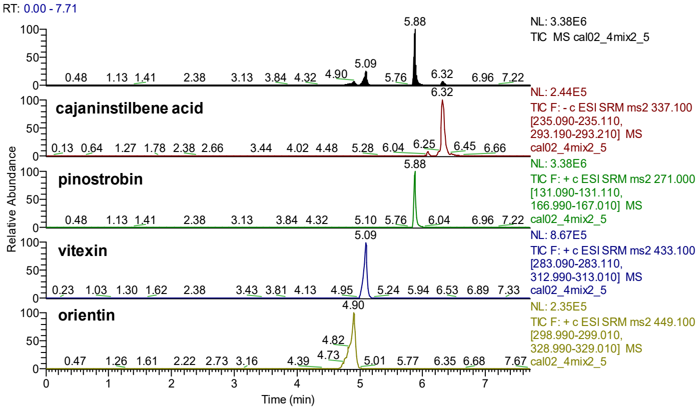

(e)

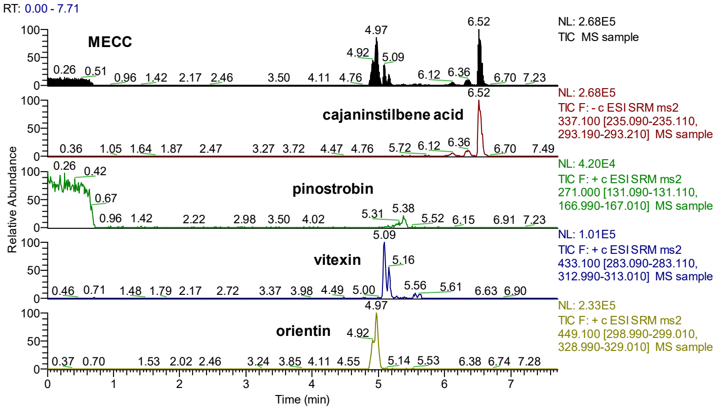

(f)

Figure S1. Chemical structure of (**a**) cajaninstilbene acid (**b**) pinostrobin (**c**) vitexin (**d**) orientin. (**e**) The multiple reaction monitoring (MRM) chromatograms of standard solution with 2.5 μM of cajaninstilbene acid, pinostrobin, vitexin and orientin. (**f**) The MRM chromatograms of 10 μL MECC sample (50 mg/mL).
